# Puerarin inhibits vascular smooth muscle cells proliferation induced by fine particulate matter via suppressing of the p38 MAPK signaling pathway

**DOI:** 10.1186/s12906-018-2206-9

**Published:** 2018-05-04

**Authors:** Qiang Wan, Zhongyong Liu, Yuping Yang

**Affiliations:** 1 Department of Medical Cardiology, the Affiliated Hospital of Jiangxi University of Traditional Chinese Medicine, Nanchang, 330006 China; 2 Respiratory Medicine, the Affiliated Hospital of Jiangxi University of Traditional Chinese Medicine, Nanchang, 330006 China

**Keywords:** Puerarin, Vascular smooth muscle cells, Proliferation, Fine particulate matter, p38 mitogen-activated protein kinase

## Abstract

**Background:**

Fine particulate matter (PM2.5) is a major risk factor for the development and progression of atherosclerosis. Proliferation and infiltration of vascular smooth muscle cells (VSMCs) from the blood vessel media into the intima is a crucial step in the pathophysiology of atherosclerosis. Puerarin, a natural extract from *Radix Puerariae*, possesses significant anti-atherosclerosis properties. However, the underlying molecular mechanisms responsible for the effect of puerarin on the VSMCs proliferation induced by PM2.5 remain unclear. The present study was designed to examine the effect of puerarin on PM2.5-induced VSMCs proliferation, and to explore the p38 mitogen-activated protein kinase (p38 MAPK) signal mechanism involved.

**Methods:**

VSMCs viability was measured by CCK-8 assay, VSMCs proliferation was assessed by BrdU immunofluorescence, the levels of superoxide dismutase (SOD) and malonaldehyde (MDA) were assayed by colorimetric assay kits, the levels of nitric oxide (NO) and endothelin-1 (ET-1) were determined by nitrate reductase method and radioimmunoassay, the levels of vascular cell adhesion molecule-1 (VCAM-1), interleukin-6 (IL-6) and tumor necrosis factor-alpha (TNF-α) were measured by ELISA. The protein expressions of phospho-p38 MAPK (p-p38 MAPK) and proliferating cell nuclear antigen (PCNA) in the VSMCs were subjected by Western blot.

**Results:**

Compared to the PM2.5-treated cells, in addition to inhibiting the PM2.5-induced VSMCs proliferation, puerarin also down-regulated the protein expressions of p-p38 MAPK and PCNA, decreased the levels of ET-1, VCAM-1, IL-6, TNF-α and MDA, increased the levels of NO and SOD. Moreover, the anti-proliferative effects of puerarin were significantly enhanced by the co-incubation of puerarin with SB203580, a selective inhibitor of p38 MAPK, as compared to the puerarin-treated cells.

**Conclusion:**

These results suggest that puerarin might suppress the PM2.5-induced VSMCs proliferation via the inhibition of the p38 MAPK signaling pathway.

## Background

Particulate matter less than 2.5 μm in diameter (PM2.5) air pollution exposure is associated with overall mortality, cardiovascular mortality, and cardiovascular disease (CVD) events, in particular, long-term exposure to high concentrations of PM2.5 has been associated closely with risk of atherosclerosis, the underlying pathology for CVD [1]. An air pollution review completed by the American Heart Association indicated that the inhalation of PM2.5 accelerates the development of atherosclerosis, and triggers clinical ischemic events [2]. Results from a Heinz Nixdorf Recall Study show that 1-year residential exposure to PM2.5 in the general population is positively associated with carotid intima-media thickness, which is an important index of subclinical atherosclerosis and provides a means to assess the progression and development of atherosclerotic vascular disease [3]. Additionally, an in vivo study demonstrated that PM2.5 exposure could induce considerable oxidative stress and systemic inflammation and contribute to the progression of atherosclerosis in apolipoprotein E knockout mice [4]. Although previous studies have shown associations between PM exposure and atherosclerosis, the mechanisms of PM2.5-induced atherogenesis have not been fully elucidated.

A pivotal pathogenesis of atherosclerosis and restenosis is characterized by accumulation of vascular smooth muscle cells (VSMCs) within the intima. The homeostatic balance between VSMC growth and death is important for vascular remodeling, atherosclerotic plaque formation, and its vulnerability [5]. Hence, excessive proliferation of VSMCs has been increasingly recognized as an essential contributor to the development of atherosclerotic process. Moreover, an in vitro study showed that PM extract stimulated VSMCs proliferation via the activation of extracellular signal-regulated protein kinase 1 and 2 (ERK1/2) and nuclear factor kappa B (NF-κB) pathways [6]. Evidence from previous studies has contributed to the hypothesis that PM-induced VSMCs proliferation is an essential reason in the pathogenesis of atherosclerosis.

Phytochemicals are plant-derived small molecules that possess cardio-protective effects [7–9]. Among these compounds, puerarin is the most abundant isoflavone-C-glucoside compound isolated from *Radix Puerariae*, the dried root of the leguminous plant *Pueraria lobata (Willd.) Ohwi* which is an edible vegetable in China, has been widely used for the treatment of CVD, diabetes, osteonecrosis and neurodegradation diseases [10]. Puerarin plays a positive role in improving immunity, dilating blood vessels, enhancing myocardial contractility, boosting microcirculation, lowering blood pressure, and protection myocardial cell [11]. Furthermore, several reports have suggested that puerarin possesses protective effects in atherosclerosis, its biological activities may be related to anti-oxidative, anti-inflammatory, and anti-hypercholesterolemic properties [12, 13]. Although beneficial effects of puerarin on CVD have been suggested, however, the underlying molecular mechanisms responsible for the amelioration of atherosclerosis, especially the effect on the proliferation of PM2.5-induced VSMCs, remain elusive.

The recent studies above led us to hypothesize that puerarin would display a significant inhibitory effect against PM2.5-induced VSMCs proliferation. Therefore, we investigated the effects and the underlying mechanisms of puerarin on mediating PM2.5-induced VSMCs proliferation in the present study. Understanding the intracellular signal mechanism that leads puerarin treatment to mediating PM2.5-induced VSMCs proliferation may provide novel options for the treatment of PM2.5-related atherosclerosis.

## Methods

### Reagents

**Fig. 1:**
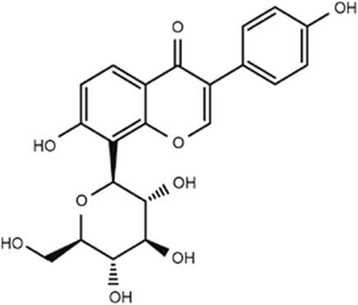
Chemical structure of puerarin

Puerarin (Fig. 1, purity: ≥ 98%) and SB203580 were purchased from Sigma-Aldrich (St Louis, MO, USA). Dulbecco’s modified Eagle’s medium (DMEM) was purchased from Invitrogen (Carlsbad, CA, USA). Fetal bovine serum (FBS) was purchased from Gibco (Grand Island, NY, USA). Interleukin-6 (IL-6), tumor necrosis factor-alpha (TNF-α) and vascular cell adhesion molecule-1 (VCAM-1) enzyme-linked immunosorbent assay (ELISA) kits were purchased from eBioscience (San Diego, CA, USA). Cell Counting Kit-8 (CCK-8), nitric oxide (NO), endothelin-1 (ET-1), superoxide dismutase (SOD) and malonaldehyde (MDA) kits were purchased from Jiancheng Bioengineering Institute (Nanjing, China). BCA protein assay kit was purchased from Beyotime (Shanghai, China). Proliferating cell nuclear antigen (PCNA), total-p38 mitogen-activated protein kinase (t-p38 MAPK), phospho-p38 MAPK (p-p38 MAPK) and β-actin antibodies were purchased from Cell Signaling Technology (Beverly, MA, USA), Bromodeoxyuridine (BrdU) cell proliferation detection kit was purchased from KeyGen BioTECH (Nanjing, China), BrdU secondary antibody was purchased from Abcam (Cambridge, UK).

### Collection and preparation of PM2.5

As previously described [14], PM2.5 samples were prepared with a slight modification. Briefly, PM2.5 samples were collected on Zefluor PTFE membrane filters (3 μm, Pall Life Sciences, USA) using a low-volume particle samplers (24 L/min, Beijing Geology Device Company, China) for 12 h (8:00 ~ 20:00) at the 4th Ring Road, which is a major artery around the centre of Beijing from January 1st to March 31th in 2016. Filters were combined to form a pooled sample at the end of sampling. PM2.5 samples were extracted from the filters by soaking for 30 min in ultra-pure milli-Q water followed by sonication for 30 min. The extracts were concentrated in a rotary evaporator, filtered through a Teflon membrane (0.5 μm) and kept protected from light at − 20 °C to maintain chemical stability until assayed.

### PM2.5 source apportionment

Chemical components were detected based on inductively coupled plasma mass spectrometry (ICP-MS; Thermo Fisher, NJ, USA) and inductively coupled plasma-atomic emission spectrometry (ICP-AES; Thermo Fisher, NJ, USA), respectively. Ion chromatography (IC) was used to measure the cations (Mg^2+^, Ca^2+^, K^+^, Na^+^ and NH_4_^+^) and the anions (SO_4_^2−^, NO_2_^−^ and Cl^−^) contained in PM2.5. The details of the method were reported as previously described [15].

### Cell culture and treatment

Human aortic vascular smooth muscle cells (HA-VSMCs) were purchased from the Chinese Academy of Sciences Cell Bank (Shanghai, China). VSMCs were cultured in DMEM supplemented with 10% FBS and antibiotics (100 U/mL of penicillin and 100 μg/mL of streptomycin) at 37 °C in a 5% CO_2_ incubator. In order to evaluate the VSMCs proliferation induced by PM2.5 or the VSMCs injury induced by puerarin, cells at a confluence of approximately 80% were stimulated with different concentrations of PM2.5 (0, 25, 50, 100, 200, 400 mg/L) for 24 h, or with 200 mg/L PM2.5 for the different time points (0, 3, 6, 12, 24, 48 h), or with different concentrations of puerarin (0, 5, 10, 25, 50, 100 μM) for 24 h, respectively. To further elucidate the effect and the potential mechanism of puerarin on PM2.5-induced VSMCs proliferation, cells were pre-treated with puerarin at different concentrations (0, 12.5, 25, 50 μM) or p38 MAPK inhibitor SB203580 (20 μM) [16] for 1 h and followed by the addition of PM2.5 (200 mg/L) for 24 h. Cells from passages 3 to 9 were used for the subsequent experiments.

### Determination of cell viability

Cells were seeded at a density of 1 × 10^4^ /well in 96-well plates and cultivated at 37 °C in a 5% CO_2_ incubator for 24 h. Then, medium was replaced with serum-free medium for another 24 h. PBS was added to cells as a control. After the pre-treatment described above, the medium was replaced with medium containing 10 μL CCK-8 for 2 h. Blank wells were set up that contained 10 μL CCK-8 only. Values of the absorbance (A) were detected at 540 nm using a Bio-Tek Plate Reader (BioTek Instruments, USA). Cell viability, which represents proliferation, was calculated according to the formula: Cell viability = [A (PM2.5) − A (blank)]/[A (PBS) − A (blank)].

### Cell proliferation assay

Cell proliferation was determined by the number of nuclei that were DAPI stained and marked with BrdU (final concentration of 20 M). The secondary antibody for BrdU was labelled with TRITC. Images were obtained by using a microscope (Leica Microsystems, Germany).

### Biochemical analysis

The levels of SOD and MDA were assayed by colorimetric assay kits. The level of NO in the supernatant of cultured VSMCs was evaluated by nitrate reductase method according to the manufacturer’s recommendation. ET-1 level was determined by using radioimmunoassay technique following the manufacturer’s instructions. VCAM-1, IL-6 and TNF-α levels in the culture supernatant were measured using ELISA kits according to the manufacturer’s protocol.

### Western blot analysis

The protein concentrations were quantified by BCA protein assay kit. Samples (50 μg per lane) were separated by different concentrations of SDS-polyacrylamide gel (SDS-PAGE) and were transferred to polyvinylidene difluoride (PVDF; Millipore, USA) membranes for incubation at 37 °C for 2 h with blocking solution (5% non-fat milk). Then, membranes were further incubated with 1:1000 dilution primary antibodies for PCNA, p38 MAPK or p-p38 MAPK overnight at 4 °C. Membranes were washed and incubated with a 1:2000 dilution of IgG-horseradish peroxidase-conjugated second antibody for 1 h. Proteins were visualized on Kodak 2000MM. Relative intensities of protein bands was analyzed by PDI Imageware System (Bio-Rad, USA), β-actin was used for the protein loading control.

### Statistical analysis

Statistical analyses of 3 independent experiments are presented as mean ± SEM. The significance of the differences was analyzed by one-way analysis of variance (ANOVA) followed by a Tukey’s post hoc test for multiple comparisons. A value for *P* < 0.05 is considered statistically significant.

## Results

### Source apportionment analysis

**Fig. 2:**
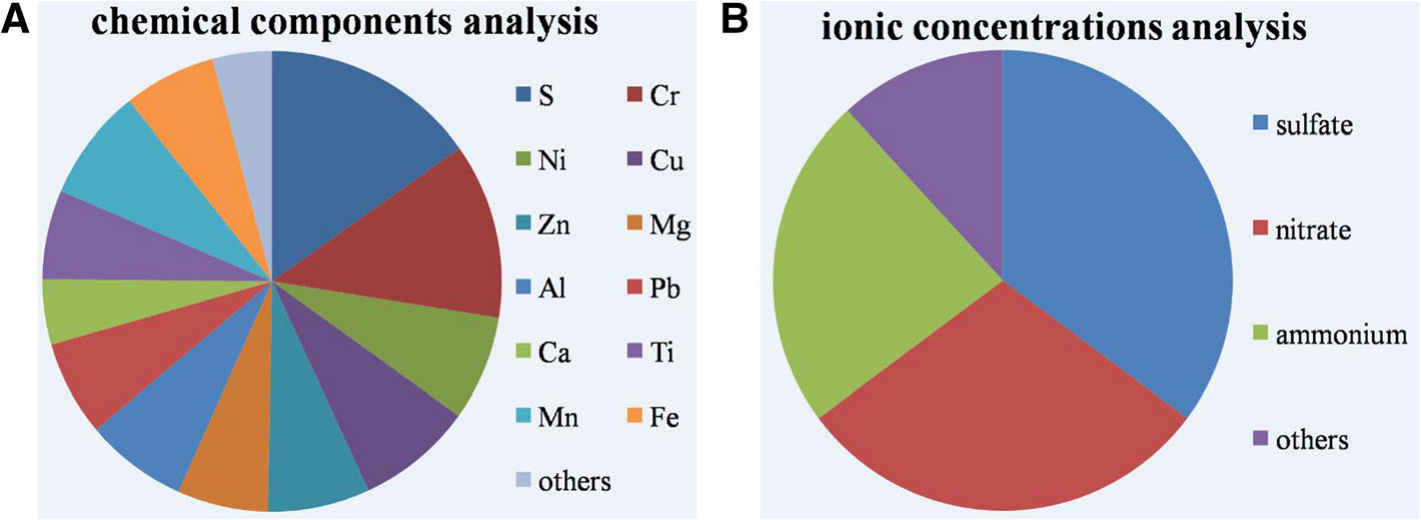
PM2.5 source apportionment analysis. Chemical components were detected based on inductively coupled plasma mass spectrometry and inductively coupled plasma-atomic emission spectrometry, respectively. **a** Chemical components analysis. **b** Ionic concentrations analysis

The chemical components analysis result (Fig. 2a) showed that S, Cu, Zn, Pb, Cr, Ni, Mg, Al, Ca, Ti, Mn and Fe were the major resources of PM2.5 pollution in Beijing. The ionic concentrations analysis result (Fig. 2b) showed that sulfate, nitrate and ammonium had the highest contribution to the PM2.5 pollution in Beijing.

### Effect of puerarin on PM2.5-induced proliferation in VSMCs

**Fig. 3:**
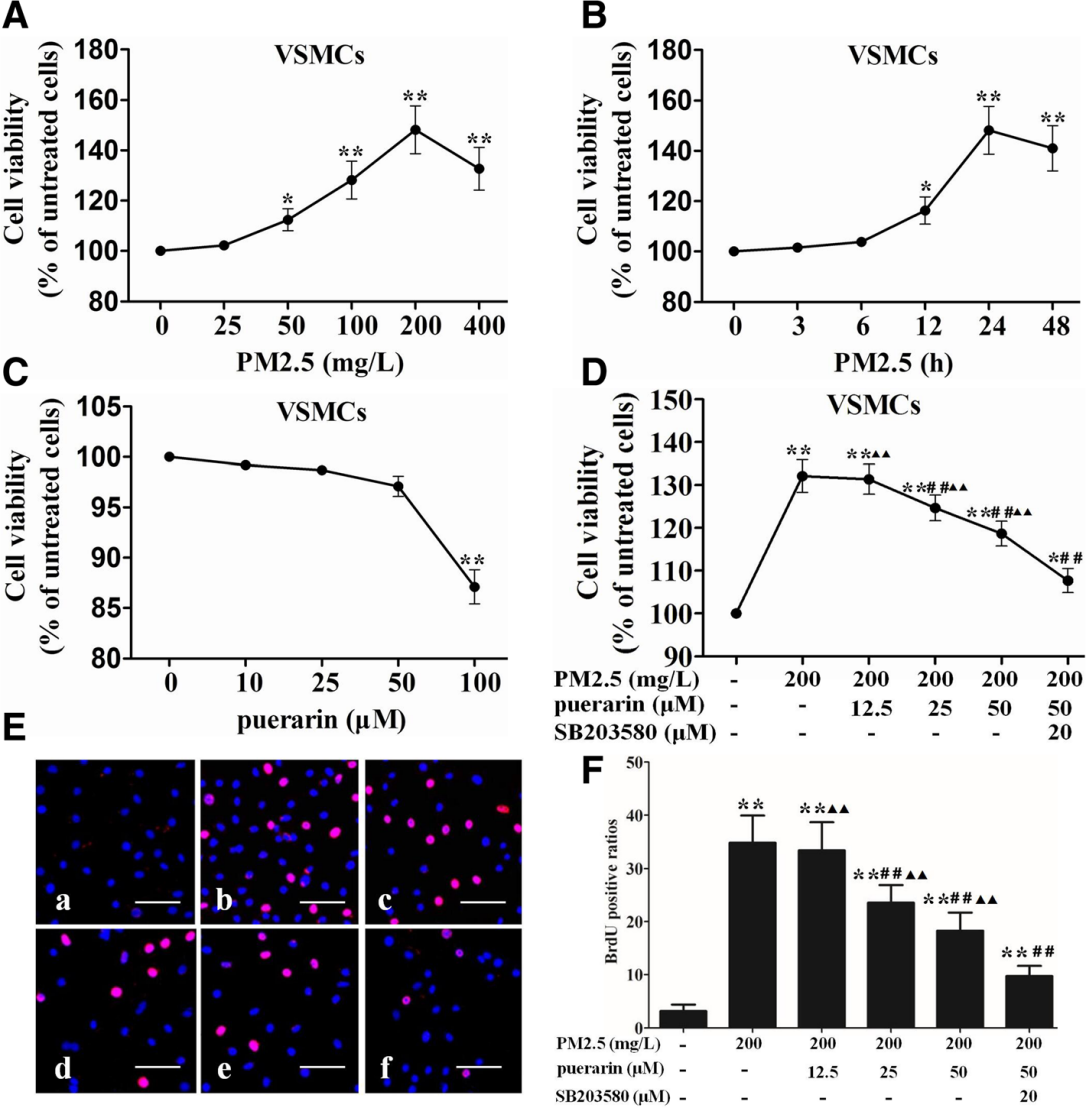
Effect of puerarin on VSMCs proliferation. VSMCs viability was assessed by CCK-8 assay. **a** Cells were stimulated with different concentrations of PM2.5 (0, 25, 50, 100, 200, 400 mg/L) for 24 h. **b** Cells were stimulated with 200 mg/L PM2.5 for the different time points (0, 3, 6, 12, 24, 48 h). **c** Cells were stimulated with different concentrations of puerarin (0, 5, 10, 25, 50, 100 μM) for 24 h. **d** Cells were pre-treated with puerarin at different concentrations (0, 12.5, 25, 50 μM) or p38 MAPK inhibitor SB203580 (20 μM) for 1 h and followed by the addition of 200 mg/L PM2.5 for 24 h. VSMCs proliferation was measured according to the BrdU immunofluorescence. **e** Confocal images. a, the untreated cells; b, cells were stimulated with 200 mg/L PM2.5 for 24 h; c, cells were pre-treated with 12.5 μM puerarin for 1 h and followed by the addition of 200 mg/L PM2.5 for 24 h; d, cells were pre-treated with 25 μM puerarin for 1 h and followed by the addition of 200 mg/L PM2.5 for 24 h; e, cells were pre-treated with 50 μM puerarin for 1 h and followed by the addition of 200 mg/L PM2.5 for 24 h; f, cells were pre-treated with 50 μM puerarin and p38 MAPK inhibitor SB203580 (20 μM) for 1 h and followed by the addition of 200 mg/L PM2.5 for 24 h. *Bars* = 100 μm. **f** Graphs of BrdU positive ratios. The results are presented as mean ± SEM. *n* = 3. Compared to the untreated cells, ^*^P < 0.05, ^**^P < 0.01; compared to 200 mg/L PM2.5 group, ^#^*P* < 0.05, ^##^*P* < 0.01; compared to the co-incubation of puerarin with SB203580 group, ^▲^*P* < 0.05, ^▲▲^*P* < 0.01

Cells proliferation was measured according to the CCK-8 assay and BrdU immunofluorescence. A concentration of 200 mg/L PM2.5 lead to significant increase in VSMCs viability (Fig. 3a). The treatment with 200 mg/L PM2.5 for 24 h led to significant increase in VSMCs viability (Fig. 3b). There was no significant difference in the viability between the puerarin treatment at 0 ~ 50 μM for 24 h as compared to untreated cells (Fig. 3c). Therefore, a treatment with 200 mg/L PM2.5 for 24 h and puerarin pre-treatment at concentration of 50 μM were considered in subsequent experiments. PM2.5 significantly increasced VSMCs viability as compared to the untreated cells, which was reversed by puerarin (25, 50 μM) administration (Fig. 3d). This anti-proliferative effect of puerarin was enhanced by SB203580 as compared to the puerarin-treated cells. The results indicated that the pro-proliferative effect of PM2.5 on VSMCs was reversed by puerarin treatment.

During the BrdU immunofluorescence, PM2.5 significantly increasced VSMCs proliferation as compared to the untreated cells. Puerarin (25, 50 μM) suppressed PM2.5-induced proliferation of VSMCs, and the anti-proliferative effect of puerarin was enhanced by SB203580 administration as compared to the puerarin-treated cells (Fig. 3e and f).

### Effect of puerarin on the levels of VCAM-1, NO and ET-1 in VSMCs

**Fig. 4:**
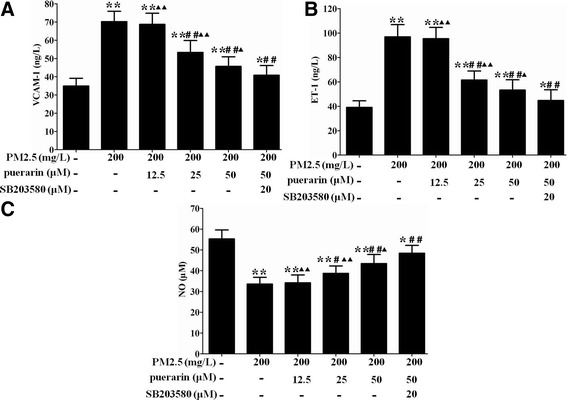
Effect of puerarin on the levels of VCAM-1, NO and ET-1 in VSMCs. Cells were pre-treated with puerarin at different concentrations (0, 12.5, 25, 50 μM) or p38 MAPK inhibitor SB203580 (20 μM) for 1 h and followed by the addition of 200 mg/L PM2.5 for 24 h. **a** VCAM-1 level was measured by ELISA. **b** ET-1 level was determined by radioimmunoassay. **c** No level was evaluated by nitrate reductase method. The results are presented as mean ± SEM. *n* = 3. Compared to the untreated cells, ^*^P < 0.05, ^**^P < 0.01; compared to 200 mg/L PM2.5 group, ^#^*P* < 0.05, ^##^*P* < 0.01; compared to the co-incubation of puerarin with SB203580 group, ^▲^*P* < 0.05, ^▲▲^*P* < 0.01

Compared with the untreated cells, PM2.5 markedly increased the levels of VCAM-1 and ET-1, and decreased the level of NO in VSMCs. Puerarin (25, 50 μM) administration significantly decreasced the levels of VCAM-1 and ET-1, and increased the level of NO in VSMCs compared with the PM2.5-induced cells. These effects of puerarin were enhanced by SB203580 as compared to the puerarin-treated cells (Fig. 4).

### Effect of puerarin on the levels of inflammatory and oxidative stress biomarkers in VSMCs

**Fig. 5:**
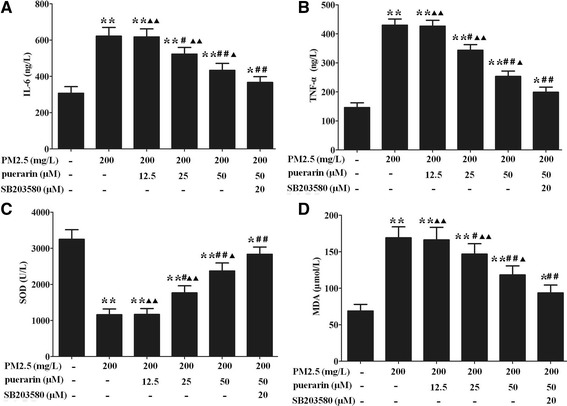
Effect of puerarin on the levels of inflammatory and oxidative stress biomarkers in VSMCs. Cells were pre-treated with puerarin at different concentrations (0, 12.5, 25, 50 μM) or p38 MAPK inhibitor SB203580 (20 μM) for 1 h and followed by the addition of 200 mg/L PM2.5 for 24 h. **a** IL-6 level was measured by ELISA. **b** TNF-α level was measured by ELISA. **c** SOD level was assayed by colorimetric assay kit. **d** MDA level was assayed by colorimetric assay kit. The results are presented as mean ± SEM. *n* = 3. Compared to the untreated cells, ^*^P < 0.05, ^**^P < 0.01; compared to 200 mg/L PM2.5 group, ^#^*P* < 0.05, ^##^*P* < 0.01; compared to the co-incubation of puerarin with SB203580 group, ^▲^*P* < 0.05, ^▲▲^*P* < 0.01

Compared with the untreated cells, PM2.5 markedly increased the levels of IL-6, TNF-α and MDA, and decreased the level of SOD in VSMCs. Puerarin (25, 50 μM) administration significantly decreasced the levels of IL-6, TNF-α and MDA, and increased the level of SOD in VSMCs compared with the PM2.5-induced cells. These effects of puerarin were enhanced by SB203580 as compared to the puerarin-treated cells (Fig. 5).

### Effect of puerarin on the expressions of p-p38 MAPK and PCNA in VSMCs

**Fig. 6:**
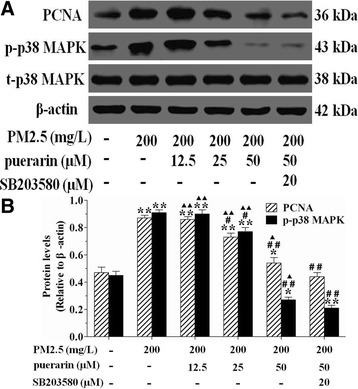
Effect of puerarin on the expressions of p-p38 MAPK and PCNA in VSMCs. Cells were pre-treated with puerarin at different concentrations (0, 12.5, 25, 50 μM) or p38 MAPK inhibitor SB203580 (20 μM) for 1 h and followed by the addition of 200 mg/L PM2.5 for 24 h. Then the expressions of PCNA, p-p38 MAPK and t-p38 MAPK in VSMCs were determined by western blot, β-actin was used for the protein loading control. **a** Representative western blot results for PCNA, p-p38 MAPK and t-p38 MAPK in VSMCs. **b** Quantitative analysis of western blot results was presented. The results are presented as mean ± SEM. *n* = 3. Compared to the untreated cells, ^*^P < 0.05, ^**^P < 0.01; compared to 200 mg/L PM2.5 group, ^#^*P* < 0.05, ^##^*P* < 0.01; compared to the co-incubation of puerarin with SB203580 group, ^▲^*P* < 0.05, ^▲▲^*P* < 0.01

Compared with the untreated cells, PM2.5 markedly increased the protein expressions of p-p38 MAPK and PCNA in VSMCs, which were reversed in cells pre-treated with puerarin (25, 50 μM). These effects of puerarin were enhanced by SB203580 as compared to the puerarin-treated cells (Fig. 6).

## Discussion

The rapid urbanization and industrialization in China have led to a sharp increase in energy consumption and pollutant emissions, especially in mega cities. Beijing, the capital of China where more than 20 million people reside, is one of the mega cities in the world with increasing number of traffic vehicles and high coal consumption during winter heating seasons (November to March). The annual consumption of coal can be as huge as 30 million tons in Beijing. The 4th Ring Road is a major artery of Beijing with 8 main lanes and 6 auxiliary lanes that carries more than 220,000 vehicles per day [17]. Evidence from source apportionment analysis indicated that gasoline exhaust and coal burning emission were the most two major resources for the total mass concentration of PM2.5 in Beijing [4]. Therefore, the collection of PM2.5 samples in this study was conducted in a building on the 4th Ring Road from January 1st to March 31th in 2016. In this study, the chemical components analysis result showed that coal combustion and metallurgy sources (S, Cu, Zn and Pb), motor vehicle exhaust (Cr and Ni) and dust sources (Mg, Al, Ca, Ti, Mn and Fe) were the major resources of PM2.5 pollution in Beijing. The ionic concentrations analysis result showed that sulfate, nitrate and ammonium had the highest contribution, indicating that the combustion of fuel with high sulfur content (such as coal and residual oil) was an important source to the PM2.5 pollution in Beijing.

Particulate matter is a complex mixture of particles with various chemical compositions including ammonium ion, elemental carbon, nitrate, organic carbon matter, silicon, sodium ion and sulfate [18]. According to its aerodynamic diameter, PM is classified into ultrafine particles (< 0.1 μm), PM2.5 (< 2.5 μm), PM10 (< 10 μm) and thoracic particles (> 10 μm). Consequently, the deleterious health effects of the particles correlate negatively with the particle size [19]. PM2.5 is very small, which makes it easier to be a greater threat to human health after entering through the trachea, traveling into the alveoli, penetrating through the pulmonary air-blood barrier, and then diffusing into the capillaries and into the systemic blood circulation [15]. Therefore, PM2.5 can directly affect the cardiovascular system and increase CVD mortality. Atherosclerosis is a progressive disease characterized by the accumulation of lipids and fibrous plaque in the arteries [20]. Its etiology is complicated and its risk factors primarily include hyperlipidemia, hypertension, genetic defects, smoking, and lack of exercise. Recent studies suggest that PM2.5 may also contribute to the development of atherosclerosis. Endothelial dysfunction was taken as the first step toward coronary arteriosclerosis, long-term exposure to PM2.5 was associated with decreased NO-mediated endothelial function in a conduit artery independent of cardiovascular risk factors [21, 22]. PM2.5 exposures in apolipoprotein E-deficient mice exhibited a significantly pro-atherogenic potential, which could be intimately linked to an inhibition of the anti-inflammatory capacity of plasma high-density lipoprotein and to a greater propensity to generate systemic oxidative stress [23].

During progression of atherosclerosis, transformation of VSMCs from the quiescent contractile phenotype towards the proliferative migratory phenotype into the plaque area to form a fibrous cap is generally regarded as a vital step in the formation of unstable atherosclerotic plaques [24]. These VSMCs that migrated into the intima exhibit an aberrantly increased proliferation and extracellular matrix production, leading to the formation of the fibrous cap in atherosclerotic lesions [25]. Endothelial NO produced by NO synthase (eNOS) induces vascular smooth muscle relaxation and suppresses the aggregation and adhesion of inflammatory cells and platelets [26]. NO also exerts multiple anti-atherosclerotic properties, including inhibition of leukocyte adhesion to vascular endothelium and leukocyte migration into the vascular wall, prevention of LDL oxidation and inhibition of VSMCs proliferation [27]. Reduced NO plays a key role in the enhanced leukocyte recruitment reflective of systemic inflammation thought to precede and underlie atherosclerotic plaque formation and instability [28]. PCNA, a nuclear protein that has been involved in amount of cellular processes including DNA replication and cell-cycle regulation, is a reliable indicator of cell proliferation status [29]. ET-1, an extremely potent and long-acting vasoconstrictor peptide originally isolated from endothelial cells, contributes to the hyperproliferation of VSMCs through the MAPK-PI3K pathway [30]. VCAM-1, an immunoglobulin-like glycoprotein, participates in the adhesion of leukocytes to endothelial cells and the subsequent transmigration to the arterial intima, promotes the VSMCs proliferation via the focal adhesion kinase pathway [31]. Several inflammatory cytokines, such as TNF-α, IL-1β and IL-6, have been shown to induce VSMCs proliferation/migration and hypertrophic response, which can contribute to the development of atherosclerosis [32]. Oxidative stress plays a pivotal role in the progression of atherosclerosis, and is involved in the regulation of VSMCs proliferation, migration and differentiation. The key antioxidant enzyme SOD inhibits neointima formation through attenuation of migration and proliferation of VSMCs [33]. In addition, MDA acts as a marker of endogenous lipid peroxidation and is formed as an end product of lipid peroxidation [34]. Mitogen-activated protein kinases (MAPKs) are a group of signaling molecules that regulate proliferation, apoptosis, differentiation and inflammatory through activating a number of downstream transcription factors [35]. p38 MAPK is known to be strongly activated in response to vascular injury, and the p38 MAPK signaling pathway has been demonstrated to be implicated in the regulation of the proliferation of VSMCs in response to proliferative factors by modulating the expression of cell cycle-associated proteins [36]. In this study, we found that PM2.5 administration significantly promoted VSMCs proliferation and markedly up-regulated the protein expressions of p-p38 MAPK and PCNA, increased the levels of ET-1, VCAM-1, IL-6, TNF-α and MDA, decreased the levels of NO and SOD. These results indicated that PM2.5 might induce VSMCs proliferation via the activation of the p38 MAPK signaling pathway.

Nowadays, the effects of puerarin in atherosclerosis are being increasingly concerned. Increasing studies have suggested that puerarin has protective effects in atherosclerosis. Puerarin had the potential to act as a protector for human umbilical vein endothelial cells against intracellular reactive oxygen species mediated apoptosis and mitochondrial damage [12]. Similarly, puerarin inhibited oxidized low density lipoprotein (ox-LDL)-induced endothelial cells injuries via the suppression of lectin-like ox-LDL receptor 1 (LOX-1) and induction of endothelial nitric oxide synthase (eNOS) [13]. The effect of puerarin on the suppression of atherosclerosis also was linked to an inhibited inflammatory response and reduced NF-κB activation [37]. Furthermore, puerarin significantly inhibited VSMCs proliferation induced by ox-LDL by suppressing PCNA expression and ERK 1/2 phosphorylation [38]. In the present study, the pro-proliferative effect of PM2.5 on VSMCs was reversed by puerarin treatment. Compared to the PM2.5-treated cells, in addition to inhibiting the PM2.5-induced VSMCs proliferation, puerarin could also down-regulate the protein expressions of p-p38 MAPK and PCNA, decrease the levels of ET-1, VCAM-1, IL-6, TNF-α and MDA, increase the levels of NO and SOD. SB203580, one of the most used pyridinyl imidazole compounds, is a selective ATP-competitive inhibitor of p38 MAPK. Compared to the puerarin-treated cells, co-incubation of puerarin with SB203580 significantly inhibited VSMCs proliferation, down-regulated the protein expressions of p-p38 MAPK and PCNA, decreased the levels of ET-1, VCAM-1, IL-6, TNF-α and MDA, increased the levels of NO and SOD. Our data suggest that puerarin might suppress the PM2.5-induced VSMCs proliferation via the inhibition of the p38 MAPK signaling pathway.

Although this study provides new insights into the understanding of VSMCs proliferation in association with PM2.5, and into puerarin’s molecular mechanism and its therapeutic potential in the treatment of PM2.5-induced atherosclerosis, other signaling pathway should not be ignored in contributing to VSMCs proliferation. Icariin was reported to reduce the amount of ox-LDL-induced proliferation of VSMCs through inhibiting of PCNA expression and suppression of the ERK1/2 signaling pathway [39]. However, whether puerarin could inhibit the PM2.5-induced VSMCs proliferation via regulating other signaling pathway, remain elusive. Further investigation is required to focus on the emerging issues from this study.

## Conclusion

The findings of this study indicate that puerarin significantly inhibits the incidence of VSMCs proliferation induced by PM2.5, the protective effect of puerarin likely resulted from the reduced levels of ET-1, VCAM-1, IL-6, TNF-α and MDA, the decreased protein expression of PCNA, and the increased levels of NO and SOD. The mechanism underlying these therapeutic effect involved inhibition of p38 MAPK signaling pathway.

## Abbreviations

CVD: Cardiovascular diseases
ET-1: Endothelin-1
IL-6: Interleukin-6
MDA: Malonaldehyde
NF-κB: Nuclear factor kappa B
NO: Nitric oxide
p38 MAPK: p38 mitogen-activated protein kinase
PCNA: Proliferating cell nuclear antigen
PM2.5: Fine particulate matter
p-p38 MAPK: phospho-p38 MAPK
SOD: Superoxide dismutase
TNF-α: Tumor necrosis factor-alpha
t-p38 MAPK: total-p38 MAPK
VCAM-1: Vascular cell adhesion molecule-1
VSMCs: Vascular smooth muscle cells
